# Detection of Ventricular Tachycardia by an Implantable Cardiac Monitor 8 Months Post-myocardial Infarction

**DOI:** 10.19102/icrm.2024.15094

**Published:** 2024-09-15

**Authors:** Mario Volpicelli, Michele Capasso, Saverio Ambrosino, Orlando Munciguerra, Antonella Laezza, Ciro Pirozzi, Luigi Sena, Francesco Terracciano, Pasquale Merone, Carlo Carbone, Luigi Nunziata, Andrea Spadaro Guerra, Daniele Giacopelli, Luigi Caliendo

**Affiliations:** 1 Cardiology Department, Ospedale Santa Maria della Pietà, Nola, Italy; 2 Emergenze Cardiovascolari, Medicina Clinica e dell’invecchiamento, Università degli studi Federico II, Naples, Italy; 3 Dipartimento di Scienze Biomediche Avanzate, Università degli Studi Federico II, Naples, Italy; 4 Clinical Unit, Biotronik Italia Spa, Milan, Italy

**Keywords:** Cardiac arrhythmia, implantable cardiac monitor, myocardial infarction, ventricular tachycardia

## Abstract

Following a non–ST-elevation myocardial infarction (MI), a 68-year-old hypertensive, severely obese woman with 45% left ventricular ejection fraction underwent an implantable cardiac monitor (ICM) insertion. After 8 months, the ICM remotely transmitted multiple non-sustained ventricular tachycardia episodes. Symptomatic during these events, the patient underwent an invasive electrophysiologic stimulation, which induced ventricular arrhythmia. Subsequently, implantable cardioverter-defibrillator implantation was recommended. Continuous remote monitoring via an ICM detected critical arrhythmias in this post-MI patient, facilitating timely intervention.

## Introduction

After a myocardial infarction (MI), the risk of sudden cardiac death (SCD) is stratified based on left ventricular ejection fraction (LVEF), with no recommended preventive measures for patients whose values exceed 35%. Nevertheless, findings from the VALsartan In Acute myocardial iNfarcTion (VALIANT) trial indicate that the incidence of SCD or cardiac arrest with resuscitation remains around 5% in patients with an LVEF of >40%, considering a 3-year follow-up period, and the discriminatory effect of LVEF declines over time.^1^

Ongoing clinical research aims to identify a high-risk subgroup among post-MI patients with mid-range LVEFs.^2^ Given the association between cardiac arrhythmias (such as atrial fibrillation, bradycardia, and ventricular tachyarrhythmia) and major complications during the post-MI period,^3^ the use of an implantable cardiac monitor (ICM) becomes particularly attractive due to its capability for remote and continuous heart rhythm monitoring. The timely identification of subclinical but serious arrhythmic events could pave the way for preventive interventions against major cardiovascular events.

In this report, we present a case involving the recording of a ventricular tachycardia (VT) episode by an ICM 8 months after insertion in a post-MI patient with a mid-range LVEF.

## Case presentation

In November 2022, a 68-year-old female patient with hypertension and severe obesity presented to our clinic with complaints of chest pain and palpitations. She was diagnosed with a non–ST-elevation MI (NSTEMI) with a troponin T peak of 654 ng/L. Diagnostic angiography revealed non-obstructive coronary arteries, with a 40% diameter stenosis in the mid-left anterior descending artery and a 50% diameter stenosis in the proximal right coronary artery. Her coronary physiology interrogation and optical coherence tomography imaging study were negative, while an electrocardiogram showed sinus rhythm with ST-segment depression. The patient was treated with medical therapy.

Forty days after the event, the patient exhibited a modest left ventricular dysfunction, with an LVEF of 45%. Additionally, she reported experiencing episodes of palpitations accompanied by dizziness. After thorough counseling with the patient, the decision was made to insert an ICM for continuous heart rhythm monitoring. The BIOMONITOR IIIm device (Biotronik SE & Co., Berlin, Germany) was inserted in the standard location as per the manufacturer’s recommendation (left pectoral region, parallel to the sternum) under local anesthesia. The patient was monitored using a remote monitoring system as part of routine clinical practice.

**Figure 1:**
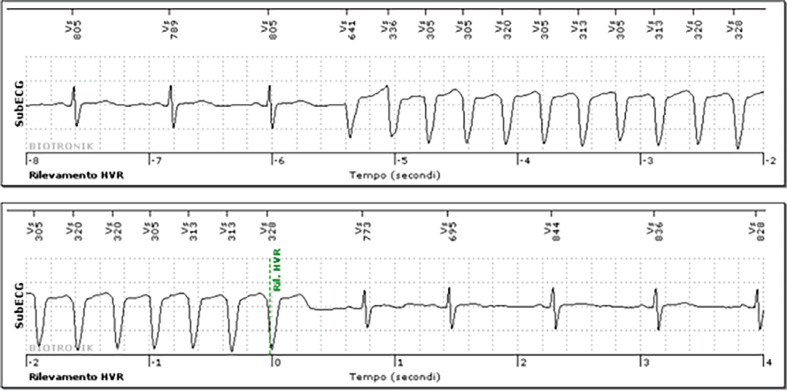
Monomorphic non-sustained ventricular tachycardia recorded and transmitted by the implantable cardioverter defibrillator. *Abbreviations:* HVR, high ventricular rate; subECG, subcutaneous electrocardiogram; Vs, sensed ventricular beat.

**Figure 2:**
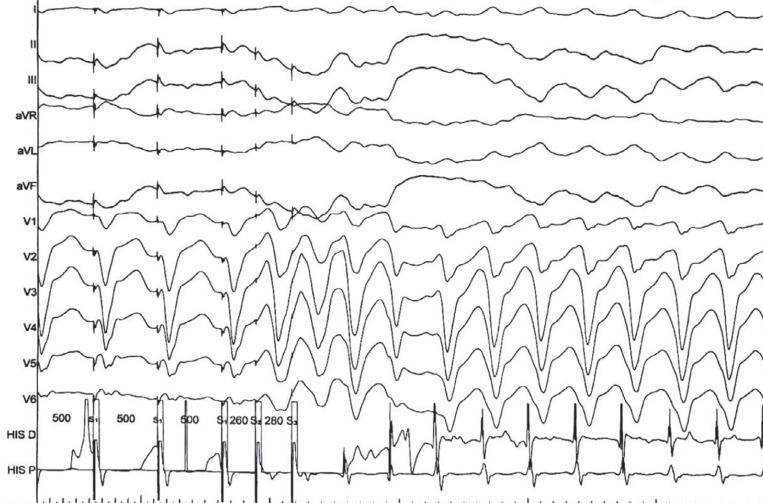
Ventricular tachycardia induced during the electrophysiologic stimulation with a basic drive cycle of 500 ms and two extrastimuli (S_1_ = 260 ms; S_2_ = 280 ms).

After 8 months of monitoring without significant arrhythmias, we received a remote transmission indicating multiple episodes of monomorphic non-sustained VT with a heart rate ranging from 180–200 bpm. A transmitted subcutaneous electrocardiogram is depicted in **Figure 1**. The patient was contacted, and she confirmed symptoms associated with these episodes. Due to the ischemic cardiomyopathy and symptoms associated with the documented arrhythmias, the patient was referred for an invasive electrophysiologic stimulation, involving two basic drive cycles (600 and 500 ms). The coupling interval of the extrastimuli was reduced in 10-ms steps until reaching a minimum of 200 ms. The electrophysiologic stimulation was positive, inducing a monomorphic VT with a cycle length of approximately 300 ms **(Figure 2)**, and the patient was indicated for implantable cardioverter-defibrillator implantation while continuing anti-arrhythmic drug therapy.

Informed consent was obtained from the patient for the publication of the case report.

## Discussion

Preventing and treating ventricular arrhythmias in the post-MI period, as well as SCD remote from the event, remain areas of research. Individuals surviving MI face a persistent high risk of mortality even years after leaving the hospital. Long-term mortality rates are comparable between NSTEMI and ST-elevation MI patients, exceeding 20% at 10 years.^4^ Approximately 50% of deaths in these patients result from SCD secondary to sustained VT or ventricular fibrillation.^5^ A diminished LVEF currently serves as the primary criterion in determining the necessity for prophylactic implantable cardioverter-defibrillator placement post-MI. However, a noteworthy observation is that the majority of SCD cases involve individuals with an LVEF exceeding 35%. Considering the multiple mechanisms involved in SCD, relying on a single test for risk stratification in all patients appears unlikely to be sufficient.^6^ Thus, the need of a validated combined approach incorporating clinical variables and the results of various stratification techniques is evident. Continuous heart rate monitoring may emerge as one of these components. Cardiac magnetic resonance represents another valuable tool with significant potential for risk stratification. In our case report, the absence of this examination prior to ICM insertion represents a limitation.

The Cardiac Arrhythmias and Risk Stratification After Acute Myocardial Infarction (CARISMA) study initially explored the use of an ICM in post-MI patients to promptly detect and treat cardiac arrhythmias.^3^ In this study, 297 patients with an acute LVEF of ≤40% received an ICM within 11 ± 5 days after MI and were monitored for 1.9 ± 0.5 years. Bradyarrhythmias and tachyarrhythmias were prevalent, recorded in 46% of patients, with 86% being asymptomatic. A recent CARISMA substudy revealed an increased long-term risk of arrhythmia and subsequent major cardiovascular events in non-revascularized patients compared to those who underwent primary percutaneous intervention.^7^

Building on CARISMA, The Implantable Cardiac Monitors in High-risk Post-infarction Patients with Cardiac Autonomic Dysfunction and Moderately Reduced Left Ventricular Ejection Fraction (SMART-MI-DZHK9) trial randomized 400 MI survivors with an LVEF between 36%–50% to ICM or conventional follow-up.^8^ Over a median follow-up of 21 months, the ICM group exhibited a significantly greater detection rate of arrhythmic events (30%) compared to the control group (6%), including atrial fibrillation, bradycardias, and VT. These findings reinforced the effectiveness of ICM in early arrhythmia detection among post-MI patients with a mid-range LVEF.

Our case involved the recording of VT episodes 8 months after ICM insertion in an NSTEMI patient with a non-critical coronary stenosis. Notably, in the CARISMA study, 45 patients received prophylactic cardioverter-defibrillators due to non-sustained VT recorded by the ICM. The Metabolic Efficiency with Ranolazine for Less Ischemia in Non–ST-Elevation Acute Coronary Syndromes—Thrombolysis In Myocardial Infarction 36 (MERLIN-TIMI36) trial, including 6560 NSTEMI patients, demonstrated that non-sustained VT over four beats was associated with an increased risk of SCD at 1 year.^9^ This case report underscores the observation that patients post-MI with an LVEF of >35% can exhibit both non-sustained and sustained VT. Throughout follow-up, a meticulous evaluation of symptoms indicative of ventricular and atrial arrhythmias becomes crucial. However, symptomatology may occasionally be non-specific or challenging to assess. In select cases, the use of an ICM may prove to be a valuable approach for detecting cardiac arrhythmias in this population, facilitating timely medical intervention. However, the translation of these findings into demonstrable improvements in clinical outcomes has not been established and requires further exploration. The Biomonitoring in Patients with Preserved Left Ventricular Function After Diagnosed Myocardial Infarction (BIO-GUARD-MI) trial aimed to elucidate whether early detection and treatment of cardiac arrhythmias using an ICM in post-MI patients can reduce the incidence of major adverse cardiac events.^2^ The trial was prematurely halted, revealing a trend of decreased incidence in the primary endpoint, although statistical significance was not achieved. A post-hoc subanalysis indicated that NSTEMI patients who underwent ICM placement were approximately 30% less likely to encounter the primary endpoint.^10^ These additional data are expected to stimulate further research into the role of continuous heart rhythm monitoring post-MI.
